# Psychosocial Correlates among Adolescents of Responses to HIV Prevention Interventions

**DOI:** 10.1155/2012/864683

**Published:** 2012-08-15

**Authors:** Bonita Stanton, Susan Rosenthal, Xiaoming Li

**Affiliations:** ^1^Wayne State University School of Medicine, Detroit, MI 48201, USA; ^2^Columbia University School of Medicine, New York, NY 10027, USA

In our request for papers exploring psychosocial issues contributing to or influencing the impact of HIV prevention interventions, we anticipated a wide range of experimental and analytic approaches and a diversity of target populations. In this regard, we were not surprised. What we did not anticipate was the geographic reach of interest in this topic. Among the nine manuscripts comprising this special issue, research was conducted in six nations from four continents. 

Represented in this issue is research conducted in Africa (see paper by K. Michielsen et al.), The Bahamas (see paper by V. Dinaj-Koci et al.), India (see paper by S. Kurapati et al.), The Philippines (see paper by L. Urada et al.), Vietnam (see paper by V. Pham et al.), and the United States (see papers by G. L. Ream et al., J. M. Sales et al., A. Metzger et al., and E. Baumler et al.). The global representation in this special issue is appropriate in that the HIV risk for adolescents does not occur primarily in one or two nations; it is a global epidemic. 

Target populations are similarly varied and include adolescents in schools, youth self-identifying as “at risk,” and youth in high risk situations such as homelessness and commercial sex work. Across the manuscripts, the results point to the need to understand both the impact of underlying contextual vulnerabilities such as financial distress for homeless LGBT or attendance at schools with poorer academic performance as well as protective factors such as adult oversight and guidance (e.g., parental monitoring and its responsiveness to an intervention in West Virginia, the positive effect of a parent enhanced young adult intervention in Vietnam, with a similar finding in The Bahamas). This also included the willingness to look to adults that may seem counterintuitive to some, that is, partnering with managers of commercial sex organizations to reduce adolescent sex worker risk exposure in The Philippines. As has often been the case, these studies underscore the importance of perceived self-efficacy in intervention effect, while perspectives on the role of knowledge and cognition varied somewhat, with affirmation in a study in the United States but uncertainty as to its role in a meta-analysis conducted in Africa.

 Taken together, these diverse papers signal a sustained and growing global commitment to understanding and impacting the psychosocial factors associated with risk and protection in the worldwide HIV epidemic. Studies such as these that take novel approaches and examine mediators of intervention effectiveness will improve our ability to support and protect adolescents in general and those that are most vulnerable in particular. These studies also enable us to find commonalities in risk and protection among diverse populations to identify strategies that appear to apply to diverse settings and strategies that may not be effective among some populations or situations.

We have enjoyed the process of crafting this special issue. We are excited that each of the individual papers is of very high quality and that the issue as a whole advances our understanding of adolescent HIV risk and prevention.

*Bonita Stanton* *Susan Rosenthal* *Xiaoming Li*

